# Sorafenib in Advanced Hepatocellular Carcinoma: A Nationwide Retrospective Study of Efficacy and Tolerability

**DOI:** 10.1155/2013/931972

**Published:** 2013-01-29

**Authors:** Anne Helene Køstner, Morten Sorensen, René Krøjgaard Olesen, Henning Grønbæk, Ulrik Lassen, Morten Ladekarl

**Affiliations:** ^1^Department of Oncology, Aarhus University Hospital, Norrebrogade 44, 8000 Aarhus, Denmark; ^2^Department of Oncology, Rigshospitalet, 2100 Copenhagen, Denmark; ^3^Department of Hepatology and Gastroenterology, Aarhus University Hospital, 8000 Aarhus, Denmark

## Abstract

*Background*. Advanced HCC is a clinical challenge with limited treatment options. The multikinase inhibitor sorafenib is the first and only agent showing a survival benefit in these patients. In this study we evaluate the efficacy and tolerability of sorafenib in an unselected patient population. Furthermore we explore the role of alpha-fetoprotein (**α**FP) as a potential biomarker for treatment efficacy and correlation to survival. *Methods*. Seventy-six patients with advanced HCC, not eligible for locoregional therapy, treated with sorafenib between 2007 and 2009 were included. Followup was until 2011. *Results*. Patients in PS 0-1 had a median overall survival (mOS) of 6.2 months, compared to 1.8 months in patients with poorer PS (*P* = 0.005). Child-Pugh A patients had a mOS of 6.6 months versus 3.6 months among patients in Child-Pugh B or C (*P* = 0.0001). Serum **α**FP ≥ 200 at baseline was prognostic for a shorter survival. All patients with radiologically verified tumor response and baseline **α**FP ≥ 200 experienced a significant decline in **α**FP within the first four weeks of treatment. *Conclusion*. The survival of patients with advanced HCC treated with sorafenib is dependent on performance status and liver function. Treatment of patients with compromised liver function and poor performance status cannot be recommended. The correlation between **α**FP and objective tumor response warrants further investigation.

## 1. Introduction

Until recently treatment options for advanced or unresectable hepatocellular carcinoma (HCC) have been limited as chemotherapy in general is ineffective [1]. Significant progress in the treatment of HCC was therefore made with the approval of the multikinase inhibitor sorafenib for this indication [2]. The approval was based upon two placebo-controlled randomized trials which for the first time could demonstrate a survival benefit in HCC patients treated with sorafenib [3, 4]. The majority of patients included in these studies were in ECOG performance status (PS) 0 or 1 and had an adequate liver function classified as ChildPugh A (CP-A).

However, in clinical practice the majority of patients with advanced HCC have severe liver cirrhosis and substantial comorbidity, compromising their general medical condition and liver function [5].

Despite the lack of evidence of a survival benefit, many HCC patients with Child-Pugh B and even C liver cirrhosis are treated with sorafenib [6]. Therefore a study of the efficacy and tolerability of sorafenib in an unselected patient population was warranted. 

 Moreover, one of the challenges in the treatment with sorafenib is the difficulties in assessing tumor response by traditional response criteria. The pivotal trial by Llovet et al. reported a very modest response rate of only 2% [4]. The lack of a correlation between objectively observed response and clinical benefit complicates treatment evaluation and clinical decision making [7]. A clinical improvement in patients' symptoms may not be expected, as no significant difference between time to symptom worsening has been observed in sorafenib-treated patients compared to placebo [4]. 

Recent studies indicate that an early decline in serum *α*-fetoprotein (*α*FP) may be a predictive marker for treatment response to targeted therapies in advanced HCC [7, 8]. 

In the current study we evaluate the efficacy and tolerability of sorafenib in an unselected patient population as they present in every day clinical practice. Furthermore we explore the role of *α*FP in treatment evaluation and its correlation to survival outcome.

## 2. Materials and Methods

### 2.1. Patients

Access to sorafenib was made available in August 2007 through a program under the Danish National Board of Health. All patients considered for sorafenib were reviewed by a panel of experts appointed by the National Board of Health and referred to one of two centrs designated to treat HCC patients with sorafenib in Denmark. A common set of criteria for the selection of patients were used by the two centres: advanced hepatocellular carcinoma diagnosed according to the criteria of EASL [9], not amendable for locoregional treatment (including transcatheter arterial chemoembolization, radio frequency ablation (RFA), and surgery), ECOG PS 0–2, CP A or B, and no substantial co-morbidity (uncontrolled cardio- or cerebrovascular disease, recent bleeding episodes, or active ulcer disease).

All patients had a dynamic three-phase CT scan performed at baseline as well as an electrocardiogram, blood pressure measurement, blood samples including haematological values, liver biochemistry, and serum *α*FP.

### 2.2. Treatment

Sorafenib was administered at a dose of 800 mg daily. However, weak or elderly patients started at a reduced dose of 400 mg daily, with the possibility of dose escalation. Dose reduction and treatment delay were performed according to the recommendations in the summary of product characteristics [2].

Treatment was continued until radiological or clinical progression, unacceptable toxicity, death, or patient refusal. 

Patients were seen every 4 weeks for toxicity management and clinical assessment. 

Response evaluation was performed every 12 weeks and included a CT-scan, liver biochemistry, and serum *α*FP. 

### 2.3. Efficacy and Toxicity Assessment

Retrospectively, patients were classified as responders, if regression of tumor lesions was noted by the reading radiologist at the evaluation CT-scan compared to baseline, without the appearance of new lesions. Strict RECIST criteria were not applied as the measuring of tumor lesions had not been performed uniformly at the time of treatment.

Patients with a serum *α*FP of ≥200 ng/L at baseline and with a decline in *α*FP of ≥20% after 4 weeks of sorafenib therapy were classified as *α*FP responders.

Toxicity was assessed based on information noted in the medical records and graded according to NCI-CTCAE v3.0 [10].

Comorbidity was scored according to the Charlson comorbidity classification [11].

### 2.4. Statistical Analysis

The primary end points were overall survival (OS) (death from all causes) and time on treatment (TOT). The analysis of objective tumor response was performed according to an intention to treat analysis (ITT) as well as an analysis of evaluable patients only, that is, patients treated for at least 12 weeks. 

The Kaplan-Meier method was used for the survival analysis. A Cox proportional hazard analysis of baseline patient and disease-specific characteristics was performed to assess a potential correlation to survival outcome and followed by a multivariate Cox regression analysis.

The level of statistical significance was 5%. All *P* values are two sided and reported with 95% confidence intervals. All statistical analyses were carried out using SPSS software.

## 3. Results

### 3.1. Patient Characteristics

A total of seventy-six patients were consecutively treated at the Departments of Oncology at Rigshospitalet, Copenhagen, and at Aarhus University Hospital, Aarhus, Denmark, between August 2007 and April 2009 and followed until 2011. Median follow-up time was 6.3 months, ranging from 4 to 777 days.

Table 1 shows baseline patient and disease characteristics together with the results of the univariate survival analysis of potential prognostic factors. Seventy-eight per cent of the patients were in PS 0-1, and 57% had a well preserved liver function (CP-A). Alcohol was the primary cause of liver disease, followed by HCV and HBV. A large proportion of patients had highly advanced disease with macroscopic vascular invasion (46%) and extrahepatic metastases (43%). The majority of patients (76%) suffered from one or more serious comorbid disorders with the most frequent being cardiovascular disease and diabetes mellitus.

### 3.2. Treatment Outcome

**Figure 1:**
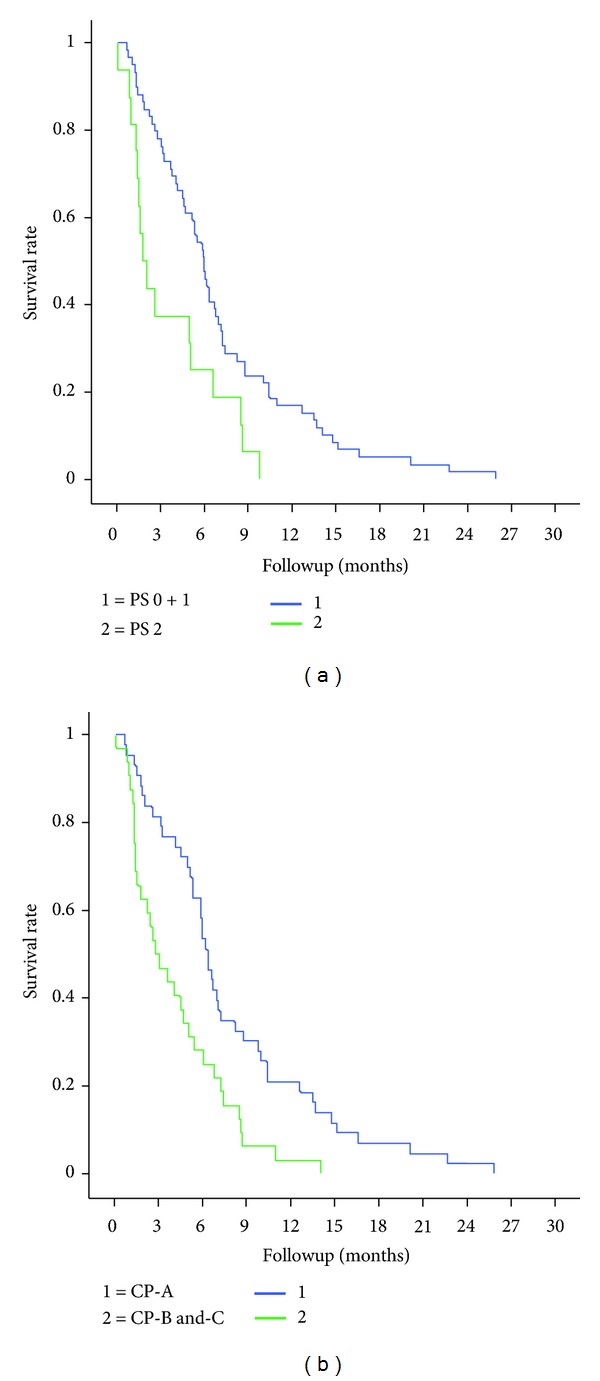
Kaplan-Meier curves for overall survival (OS) in sorafenib-treated HCC patients, stratified for (a) performance status (PS) (*P* = 0.005) and (b) Child-Pugh (CP) Class (*P* < 0.001).

The median OS (mOS) for the entire cohort of patients was 5.4 months, ranging from 4 days to more than 777 days. As illustrated in Figure 1, patients in PS 0-1 had a mOS of 6.2 months, whereas patients in PS 2-3 had a mOS of 1.8 months (*P* = 0.005). CP-A patients had a mOS of 6.6 months versus 3.6 months among CP-B and CP-C patients (*P* < 0.001).

The median time on treatment (mTOT) was 2.9 months, ranging from 4 to more than 646 days. Time on treatment was highly correlated to PS with patients in PS 0-1 being treated more than twice as long as patients in PS 2-3 (3 versus 1.4 months, *P* = 0.005). Likewise, patients in CP-A had a mTOT of 3.2 months compared to 1.5 months among patients in CP-B or -C (*P* = 0.001). 

Beside PS and Child-Pugh status, baseline albumin and bilirubin levels had significant influence on survival in the univariate analysis. Rash of any grade observed during sorafenib treatment tended to be a favorable prognostic parameter, but, not statistically significant (mOS 7.8 versus 6.7 months, *P* = 0.183).

The multivariate analysis showed that only PS and baseline albumin had independent prognostic value (*P* = 0.033 and 0.045, resp.).

Fifty-one per cent of the patients did not receive a full dose of sorafenib, either because of reduced dosing at the initiation of therapy or because of dose reduction during treatment. The mean daily dose was 539 mg of sorafenib. There was a trend that patients receiving sorafenib in a reduced dose had a shorter survival compared to the patients treated with a full dose (mOS 3.2 versus 6.2 months, *P* = 0.063). Twenty-six per cent of the patients discontinued sorafenib therapy during the first 4 weeks. Discontinuation of treatment was due to objective disease progression (24%), symptomatic progression (22%), or general deterioration (22%). Only three patients (4%) stopped sorafenib therapy due to a specific adverse event. Five patients died while on treatment, all of them due to disease progression. Nine patients were still on treatment at the end of followup.

Thirty-four patients (45%) completed at least 12 weeks of sorafenib therapy and were evaluable for assessment of tumor response according to the definition sited above (Table 2). There were no complete responders. Seven patients (9%) had a partial response with substantial regression of tumor lesions on the CT scan. All responders were in PS 0-1 at baseline, and 5 of the total 7 were classified as CP-A.

Thirty-four per cent of the patients had a serum *α*FP ≥200 ng/L at baseline. These patients had a significant poorer survival compared to patients with *α*FP <200 ng/L (*P* = 0.016). Twelve of the patients with *α*FP ≥200 ng/L at baseline experienced a decline in *α*FP of ≥20% at week 4. The survival of these patients was not significantly different from the patients without a decline in *α*FP. However, all patients with radiologically verified tumor response experienced a decline in *α*FP within the first 4 weeks of sorafenib therapy.

### 3.3. Toxicity

Thirty-three per cent of the patients experienced a grade 3-4 toxicity, with the most frequent being fatigue, diarrhoea, and hand-foot syndrome. Hypertension of any grade was seen in 18% of the patients (Table 3).

## 4. Discussion

Despite significant progress with the advent of sorafenib as a treatment option for advanced HCC, this disease is still a great clinical challenge. 

In this retrospective, comprehensive population of sorafenib-treated HCC patients, we found an overall median survival of only 5.4 months. This survival rate is considerably lower than that in the SHARP trial (10.7 months), and to some extent also in the Asian-Pacific trial (6.5 months) [3, 4]. 

We found that the prognosis was strongly dependent on both performance status and liver function. Patients with a favourable performance and an adequate liver function were both treated and lived almost twice as long as the more compromised patients, but still not as long as the patients in the randomized approval studies. This may be explained by the different characteristics of the patients included in our study compared to the patients included in the SHARP and Asian-Pacific trials. In the present study a large proportion of the patients were in PS 2 and even 3, and they, to a larger extent, suffered from a compromised liver function. Furthermore the aetiology of liver disease also differed with the majority of patients having alcohol related liver disease, whereas only about 20% was HBV or HCV positive. In contrast, in the SHARP and the Asian-Pacific trials, respectively, 50 and 70% had virus-associated HCC. Patients with HCC and a history of alcohol abuse may be especially prone to comorbid disorders which negatively influence the effect and tolerability of sorafenib. Hence, seventy-six per cent of the patients included in our study had a diagnosis of at least one serious comorbid disorder, and half of the patients did not receive a full dose of sorafenib. A more recent, prospective study of 34 patients classified as CP-B or -C treated with sorafenib reported a median OS of 3.4 months, which is close to the survival rate we found in this study (mOS of 3.6 months for CP-B patients) [12]. 

In contrast to this, 9 patients in our study turned out to be long-term survivors and were still on treatment at the end of followup, suggesting that sorafenib in some patients may be exceptionally effective, and case reports of complete responders have been published [13, 14]. Reliable molecular predictive factors, enabling the identification of such patients, are therefore greatly needed. 

Alpha-fetoprotein (*α*FP), a paraprotein released from about 70% of all hepatocellular carcinomas, has previously been suggested as a surrogate marker for treatment response in HCC [7]. In agreement with larger studies we found that elevated *α*FP at baseline was a negative prognostic factor [8]. Patients classified as *α*FP responders, that is, patients with a significant decline in *α*FP of ≥20% after four weeks of therapy, tended to have an improved survival compared to patients with an unchanged or rising *α*FP. The difference was not statistically significant, though, probably due to the small number of patients. However, of particular interest, all patients with objective tumor response and elevated *α*FP at baseline experienced a significant decline in *α*FP within the first four weeks of therapy. This calls for further investigation of *α*FP as an early biomarker for treatment response to sorafenib therapy. 

The toxicity profile of sorafenib in our study is similar to what has been reported earlier [3, 12, 15, 16]. As shown in previous studies [12], sorafenib is generally tolerable also in the more compromised patients as the number and grade of adverse events did not differ significantly among the patients with good versus poor PS and liver function. However, it should be noted that the poorer patients received sorafenib for a significant shorter period and were more often dose reduced compared to the more fit patients.

In conclusion, sorafenib treatment is feasible and generally well tolerated in HCC patients with favourable PS and Child-Pugh status. The survival of patients with compromised PS or inadequate liver function is extremely poor, even when treated. Therefore sorafenib treatment in these individuals cannot be recommended. The correlation between an early decline in *α*FP and objective tumor response suggests *α*FP as a biomarker for treatment efficacy, which should be investigated further in future clinical trials. 

## Figures and Tables

**Table 1:** Baseline patient and tumor characteristics in sorafenib treated HCC patients with corresponding median overall survival (mOS, days), 95% confidence intervals (CI), and *P* values of the univariate analysis.

Characteristic	No. (%)	mOS (days)	95% CI	*P* value
Gender				
Male	59 (78)	150	112–188	0.207
Female	17 (22)	209	185–233	
Age (median years)				
>63	31 (41)	176	112–239	0.799
≤63	45 (59)	153	117–188	
ECOG performance status				
0-1	59 (77)	186	57–170	0.005
2-3	16 (23)	54	20–109	
Child-Pugh Class				
A	43 (57)	191	168–214	<0.001
B	29 (38)	110	40–180	
C	4 (5)	41	0–100	
Portal vein thrombosis				
Present	32 (42)	198	120–232	0.967
Absent	35 (46)	170	88–194	
Unknown	9 (12)	217	138–174	
Extrahepatic spread				
Present	33 (43)	137	81–193	0.211
Absent	42 (55)	183	134–232	
Ascites				
Present	26 (34)	161	134–188	0.633
Absent	50 (66)	113	24–203	
*α*FP level (ng/mL)				
≥200	26 (34)	160	110–210	0.016
<200	56 (66)	178	122–234	
Serum albumin (g/L)				
≥35	39 (51)	186	169–203	0.003
<35	36 (49)	92	26–158	
Serum bilirubin (umol/L)				
≥22	33 (43)	98	36–160	0.005
<22	43 (67)	186	165–207	
Serum lactatdehydrogenase LDH (U/L)				
≥205	34 (50)	150	83–217	0.136
<205	34 (50)	176	142–210	
Comorbidity*				
Presence of ≥1 substantial comorbidities	58 (76)	156	127–185	0.793
No comorbidity	18 (24)	191	28–354	

*Comorbidity assessed according to Charlson comorbidity classification.

**Table 2:** Response to sorafenib treatment in patients with advanced HCC according to the intention to treat analysis (ITT) and in patients treated for at least 3 months.

Response	ITT, *N* (%)	Evaluable pt.* N(%)
Complete response	—	—
Partial response	7 (9.3)	7 (21.9)
Stable disease	18 (24.0)	18 (56.2)
Progressive disease	8 (10.7)	8 (25)
Not assessable	1 (1.3)	1 (3)
Disease control rate	33.3%	78.1%

*Response evaluated in patients completing 3 months of sorafenib therapy.

**Table 3:** Adverse events during sorafenib therapy.

Adverse event*	All grades (%)	Grades 3-4 (%)
Fatigue	68	12
Anorexia	47	7
Diarrhoea	42	11
Rash	33	4
Nausea	32	3
Hand-foot syndrome	26	12
Hypertension	18	3
Vomiting	16	3
Thrombocytopenia	5	3
Bone marrow suppression	4	—
Metabolic/laboratory	4	1
Haemorrhage	4	—

*Adverse events based on information in medical records and graded according to NCI-CTCAE v3.0.
